# Clinical application and nursing experience of the plan-do-check-act cycle in daytime varicocelectomy

**DOI:** 10.1186/s12912-024-01765-8

**Published:** 2024-02-15

**Authors:** Kang He, Jianxiong Li, Cheng Yang, Junping Wu, Biling Wu, Hui Xia

**Affiliations:** Urology Department of the Third Affiliated Hospital of Southern Medical University, 510630 Guangzhou, Guangdong People’s Republic of China

**Keywords:** PDCA, Daytime surgery, Varicocele, Nursing

## Abstract

**Background:**

Varicocele is the most prevalent correctable cause of male infertility. Currently, surgical treatment is the primary method to enhance fertility.For many young varicocele patients who have postponed surgery due to time constraints, daytime surgery is especially crucial. Thus, this study aims to investigate the clinical and nursing application value of the Plan-Do-Check-Act (PDCA) cycle in daytime varicocelectomy.

**Methods:**

Retrospective collection of clinical data was conducted on 130 patients undergoing laparoscopic varicocelectomy in the Third Affiliated Hospital of Southern Medical University, Guangzhou,China.Among them, 65 patients who underwent daytime surgery were assigned to the observation group, while 65 patients who underwent routine hospital surgeries were assigned to the control group.The former also implemented PDCA cycle management.A comparison was made between the two groups regarding hospitalization time, hospitalization costs, and patient satisfaction.

**Results:**

The observation group exhibited a shorter hospitalization time and lower hospitalization costs compared to the control group, with higher patient satisfaction and pre-discharge visual analog scale (VAS) scores noted (*P* < 0.05).No significant difference was observed in the incidence of postoperative complications between the two groups during hospitalization (*P* > 0.05). The implementation of the PDCA cycle in the observation group has demonstrated its effectiveness, ensuring the smooth conduct of the daytime varicocelectomy.

**Conclusion:**

In conclusion,daytime varicocelectomy can reduce hospitalization time,lower hospitalization costs, improve patient satisfaction. The PDCA Cycle enhances the rationality and efficacy of the daytime varicocelectomy procedure and is highly recommended. Furthermore, it offers valuable reference for the application of the PDCA Cycle in various other diseases and nursing management approaches.

**Trial Registration:**

The Trial Registration Number: ChiCTR2300077465;Date of registration: November 9, 2023.

## Background

Varicocele (VC) refers to the abnormal expansion and tortuosity of the racemose venous plexus within the spermatic cord, which causes symptoms such as scrotal swelling, pain, discomfort, and testicular dysfunction [1].In both adults and adolescents, the prevalence of varicocele is estimated to be approximately 15% [2].The VC has been listed by the World Health Organization as the main cause of male infertility [3, 4], and in clinical practice, varicocelectomy is currently the main method to solve the problem of spermatic vein reflux and improve fertility [5]. Laparoscopic surgery, as a routine method for treating VC, has the advantages of short surgical time, minimal trauma, and fast postoperative recovery [6],and its used widely used in clinical practice.Consequently, the primary focus of this study is on varicocelectomy.

However, given that the majority of varicocele patients are young, they often prioritize their work schedules and lack the necessary time for inpatient treatment. As a result, they often postpone seeking surgery, often leading to a worsening of their condition [7].Furthermore, with the widespread adoption of minimally invasive techniques, anesthesia recovery techniques, and the rapid rehabilitation surgery philosophy [8], the day surgery model has seen rapid development and promotion in China [9]. This is undoubtedly a favorable option for young patients with varicocele. As the name implies, day surgery involves patients undergoing surgery and being discharged within a 24-hour period, ensuring stricter control over both time and quality [8]. As a burgeoning perioperative management strategy, day surgery is characterized by its high-quality, high-efficiency, and high-benefit approach [10]. This model enhances the efficiency of healthcare resources and reduces patient costs.Adhering to the principle of “all for the patient, with the patient’s interests foremost,” day surgery has become a performance evaluation metric for hospitals in China, further propelling its widespread adoption [11].To advance the adoption of day surgery, it is imperative to foster standardization in surgical practices [9].Beyond the expertise of surgeons, nursing and educational efforts hold equal importance;hence, integrating both roles and ensuring seamless processes necessitates a robust management model.

The PDCA Cycle management, as an important way to improve management quality, mainly includes four stages: Plan, Do, Check and Act [12]. It is a scientific and standardized management method that can effectively identify the root causes of problems, establish targeted governance measures, provide continuous evaluation feedback, and achieve the goal of continuous improvement [13]. The introduction of the PDCA Cycle in clinical diagnosis, treatment, and nursing work might promote the improvement of clinical work [14].Recently, the implementation of the PDCA Cycle in surgical management has been instrumental in promoting positive outcomes.To address the issue of delayed surgical intervention in young patients due to time constraints, we have implemented a surgical workflow for daytime varicocelectomy, leveraging PDCA cycle management.This approach is particularly crucial for numerous young patients who, due to time constraints, have postponed their surgeries.Furthermore, it facilitates the advancement of day surgery, aligning with the promising trajectory of China’s healthcare services.Therefore, the aim of this study is to investigate the clinical and nursing significance of implementing the PDCA Cycle management approach in the daytime varicocelectomy process.

## Data and methods

### General information

Patients with varicocele who sourced from the Urology Department of the Third Affiliated Hospital of Southern Medical University, underwent the laparoscopic varicocelectomy between January 2021 and December 2022.The control group consisted of 65 routine inpatients, while the observation group comprised 65 daytime surgery patients managed through PDCA. The study adhered to the ethical review process for clinical trials. Clinical medical record data is kept confidential and is solely used for research purposes and not for any other purposes.

Inclusion criteria:Diagnosed with varicocele by physician;Patients who have undergone laparoscopic varicocelectomy (with surgical indications: obvious symptoms or abnormal semen quality; patients requesting surgical treatment);Patients with complete clinical data.

Exclusion criteria: Patients who combine other internal and external diseases and receive treatment simultaneously during hospitalization.

### Design

#### The plan stage

1.1 Theme selection:Patients with VC have a high incidence rate in the general male population and are considered to be the most common and correctable cause of male infertility. Approximately 21–41% of males are affected by primary infertility, while secondary infertility affects approximately 75–81% of males [6].In the context of China’s Healthy China strategy and the implementation of its open three-child policy, the treatment of infertility caused by VC holds particular significance. Patients with VC in our hospital who also have common ailments are suitable for conducting this study.

**Fig. 1:**
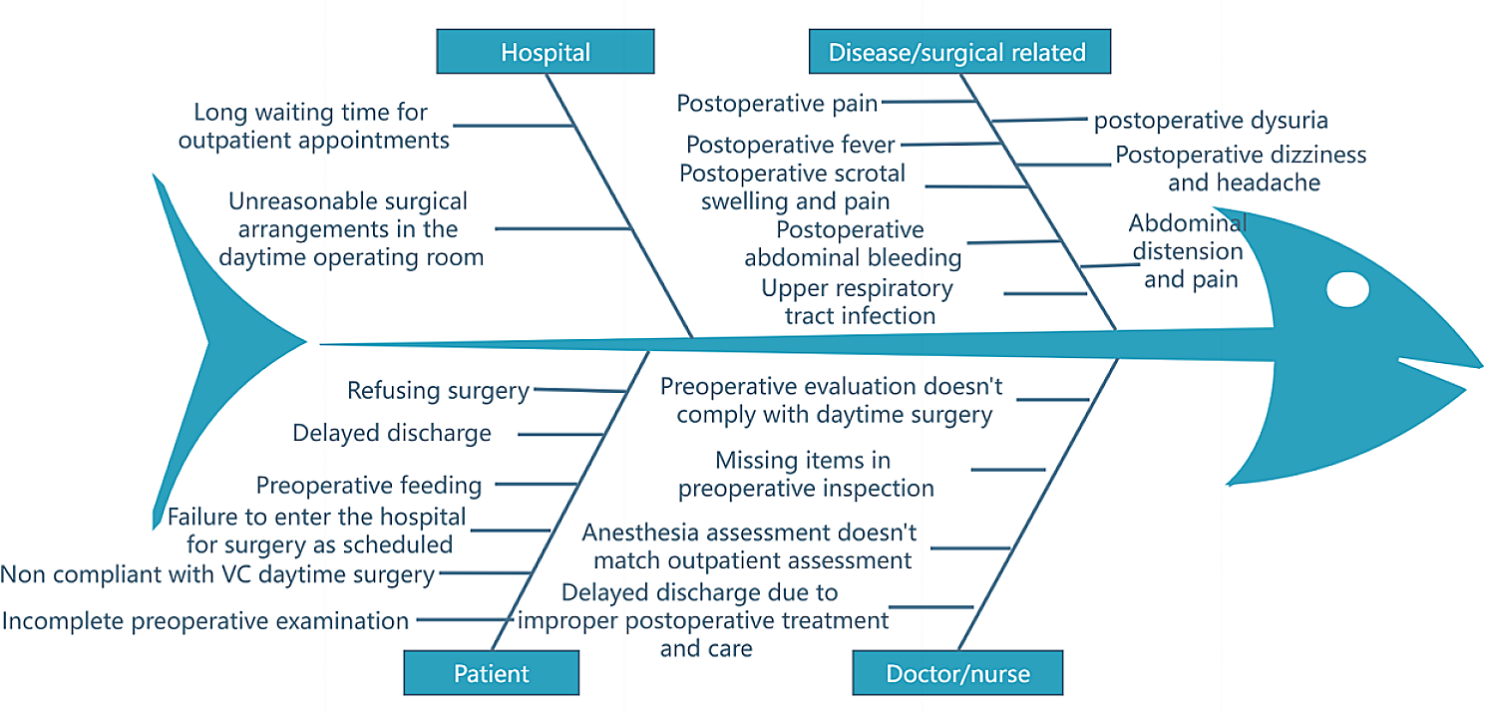
Common influencing factors of daytime varicocelectomy

1.2 Grasp the current situation and raise questions: Currently, varicocelectomy is the primary treatment for VC, as indicated by reference [15]. Laparoscopic minimally invasive technology is widely used due to its advantages of fast recovery, minimal trauma, and almost scarless treatment for patients, as stated in reference [16].Furthermore, a significant number of patients suffering from VC are young individuals who, due to their demanding work schedules, are unable to allocate substantial time for treatment in hospital, consequently causing a delay in undergoing surgery.In this mode, clinical and nursing collaboration can facilitate a swift recovery and discharge for patients, aligning with the concept of daytime surgery. Drawing from previous daytime surgery procedures, the study summarizes the pertinent factors that can influence the execution of daytime varicocelectomy. (Fig. 1) Developing this surgery process can not only shorten patients’ waiting days for surgery and hospitalization time before surgery but also reduce hospitalization costs, reduce interference in the lives of patients and relatives [9], innovate service models, improve efficiency, and meet the needs of the public for medical services.

1.3 Establish a medical and nursing working group: leader: 1 department director; deputy leader: 1 head nurse, 1 deputy chief physician; members: 3 attending physicians, 3 nursing staff.

1.4 Plan formulation: In the Plan stage,the primary objective is to select a theme, understand the current situation, analyze the root causes, and formulate a plan; In the Do stage, the plan is executed following the established process; In the Check stage, the focus is on ensuring the smooth implementation of each phase and identifying issues;In the Action stage,existing and emerging problems in each phase are addressed and improved upon.

**Fig. 2:**
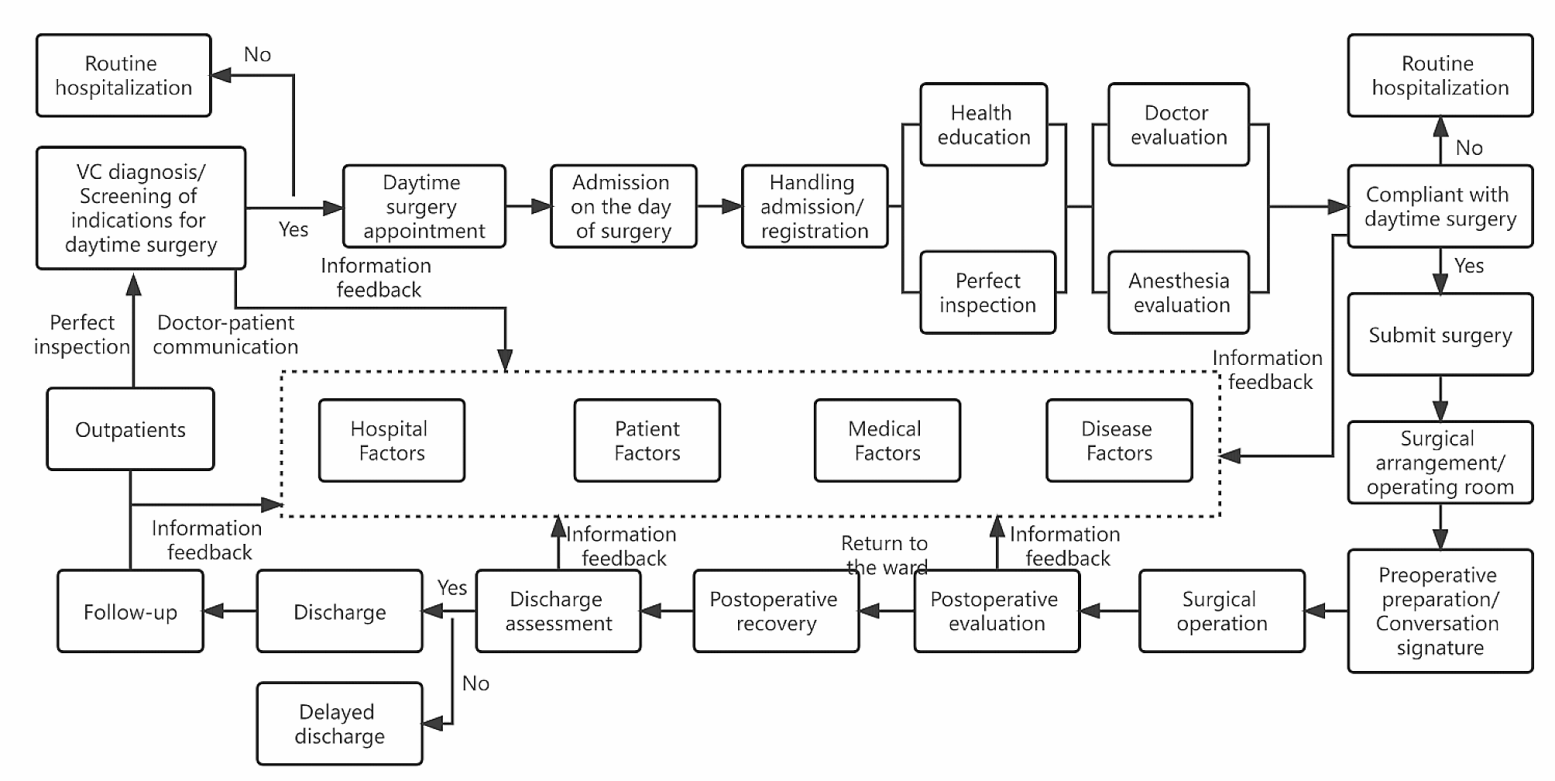
Daytime varicocelectomy procedure

1.5 Develop daytime surgery procedures: Group members will collaborate with anesthesia departments, operating rooms, imaging departments, and outpatient appointment systems to hold meetings. During these meetings, extensive thinking will take place through brainstorming methods, leading to the development of standardized daytime varicocelectomy procedures. (Fig. 2).

1.6 Evaluation indicators: (1) Hospitalization time; (2) Hospitalization cost: the total cost should be controlled within the laparoscopic surgery quota; (3) Pre discharge Visual Analog Scale (VAS) score: Select the corresponding number based on the patient’s description of pain level, and control the VAS score within 0–3 points before discharge; (4) Patient satisfaction: The overall satisfaction of patients was above 4 points. [Satisfaction score: very satisfied (5 points); satisfied (4 points); average (3 points); dissatisfied (2 points); very dissatisfied (1 point)]

### The do stage

2.1 Implement the established daytime varicocelectomy process, train team members, and familiarize them with each stage and procedure.This study strictly follows the process:

#### Outpatient and daytime surgery appointments

The patient visits the outpatient clinic where the outpatient physician conducts a preliminary diagnosis, enhances the preoperative examination (in line with the control group), evaluates whether it meets the indications for daytime varicocelectomy, and if so, schedules an appointment for daytime surgery, issues a hospitalization notice. If it fails to meet the requirements, it will be admitted to the hospital on a routine basis.

#### On the day of surgery

the admission is processed and the patient is notified in advance of fasting and water deprivation.

### Preoperative preparation

(1) The supervising nurse arranges the daytime surgery ward, registers information, and prints wristbands.Next, they educate patients on preoperative procedures, remind them of hospital precautions, perform nursing evaluations, and complete the necessary nursing documents.Lastly, they reinforce psychological and humanistic care, alleviate patients’ anxiety, and instruct them on preparing their personal belongings.

(2) The supervising physician verifies the completeness of the preoperative examination, promptly rectifies any missing items, and reevaluates the patient’s eligibility for daytime VC surgery. Simultaneously, the anesthesiologist evaluates whether anesthesia can be administered as intended. If the requirements are met, submit the surgery, contact the operating room, and arrange the operating room and table accordingly.

(3) The supervising physician signs surgical related documents, and the supervising nurse informs the patient to prepare for surgery, including preoperative urination, surgical area preparation, and transport the patient to the operating room.

#### Surgery

Operate according to the plan and wait for the patient to return to the ward after the surgery is completed.

### Postoperative care management

(1) General care: The supervising nurse provides electrocardiogram monitoring, monitors the patient’s vital signs, instructs the patient to rest in bed for 6–8 h, explains postoperative precautions, pays attention to perineal hygiene, and observes the condition of the urinary tract.

(2) Dietary care: Guiding patients to eat 8 h after surgery, starting with a semiliquid diet and gradually returning to a regular diet. It is advisable to eat small amounts and multiple meals to ensure calorie intake. Observe the recovery of intestinal function, whether there is abdominal distension and defecation.

(3) Inform the patient and his family that after 8 h of surgery, when the patient is fully awake, he can appropriately get up and move around.

(4) Postoperative medication: Follow the doctor’s instructions to use medication, observe for any adverse reactions, and take blood samples and electrolytes for reexamination after surgery.

(5) Postoperative observation of complications (video and bedside oral guidance, distribution of brochures if necessary): (i) Difficulty in urination: The supervising nurse and physician will observe and evaluate the condition of the urinary catheter, as well as monitor for any difficulty in urination or presence of hematuria after its removal. (ii) Postoperative wound condition: The supervising nurse observes whether the wound dressing has oozed blood or purulent fluid, while the supervising physician checks the patient’s blood drawing results, vital signs, and whether there is active bleeding or infection in the abdominal cavity or wound. (iii) The supervising nurse should pay attention to the hygiene of the perineum and observe whether the patient’s scrotum is swollen or there are any surgical complications such as testicular hydrocele. (iv) Pay attention to observing the patient for any anesthesia related complications such as nausea, vomiting, chest tightness, shortness of breath, dizziness, and headache. If any of the above occur, promptly report to the supervising physician.

### Discharge and follow-up

(1) Discharge: Within 24 h of admission, evaluate whether the patient meets the discharge standards. If so, the supervising physician and nurse prepare the discharge documents and change the wound dressing. Inform the patient of discharge precautions, emphasize regular follow-up, observe the wound and scrotum condition on their own, change dressing within 2–3 days, and remove the outpatient stitches one week later.

(2) Follow-up: Develop a postoperative follow-up table, which includes: one month postoperative follow-up with scrotal ultrasound to evaluate the efficacy, postoperative pain relief, postoperative wound healing, and postoperative complications; After 3, 6, 9, and 12 months of surgery, follow-up semen examination and scrotal ultrasound were conducted to evaluate the surgical efficacy. Relevant Inspection data were collected and recorded during each follow-up.

2.2 Physicians receive surgical training and assessment to shorten surgical and anesthesia time and acilitating postoperative recovery. The quality control department and medical record room cooperate with each other to develop a simplified template for the admission and discharge records of patients undergoing daytime surgery, simplify case writing, and develop a clinical pathway for daytime varicocelectomy.

### The check stage

3.1 Check the execution of the day surgery workflow, promptly addressing reasons for delayed discharge or unfinished surgeries to ensure seamless flow of the process.

3.2 Check whether the goal has been achieved by collecting the hospitalization status of patients undergoing daytime varicocelectomy and observing for any post-operative complications. Summarize the experience and analyze the feasibility and effectiveness of daytime varicocelectomy based on clinical data.

3.3 Check the implementation of the core medical system, doctor-patient communication, and informed consent of patients for the “three reasonable” situations. The head nurse implements regular and random inspections to check whether the nursing work is reasonable and standardized and whether the quality of nursing documents is up to standard; The deputy team leader verifies the existence of duplicate or redundant examinations, along with the quality of the medical documents.

### The action stage

4.1 The group holds regular meetings to solicit suggestions from team members, optimize the daytime varicocelectomy process, collect patient feedback, and continuously improve and move on to the next PDCA cycle.

4.2 Improve the sense of responsibility, enthusiasm, and initiative of medical staff through quality control and reward and punishment plans. Implement rewards and punishments based on the number of patients entering the clinical pathway and the number of qualified patients.

4.3 Through free medical consultations, short videos, popular science platforms, official accounts, and other online channels, we aim to educate men about varicocele and promote daytime varicocelectomy, ensuring they never have to worry about surgery being postponed due to time constraints.

### Statistical analysis

SPSS 25.0 software was used to process and analyze the obtained data. The econometric data are represented by the mean ± SD, with two independent sample t-tests used for inter group comparisons, and rank sum tests used for nonnormally distributed data; The counting data is represented by (N;%), using the chi-square or Fisher exact test probability method. *P* < 0.05 considered to be statistically significant.

## Results

A total of 130 clinical data of VC patients were collected, with the observation group undergoing daytime surgery and the control group undergoing routine hospitalization surgery. There was no significant difference in demographic characteristics such as age, affected side, grading, and BMI between the two groups of patients (*P* > 0.05). The majority of patients had bilateral varicocele of grade II (Table 1).

As shown in Table 2, there was a significant difference in hospitalization time between the two groups of patients (*P* < 0.05). The observation group was discharged within 24 h of admission and did not experience any return to the hospital.The hospitalization costs of the observation group were lower than those of the control group, regardless of whether the patients had unilateral or bilateral varicocele. Regarding the VAS score before discharge, the observation group had a higher score than the control group (*P* < 0.05), which may be due to the prolongation of postoperative recovery time and the use of painkillers, leading to a reduction in pain before discharge, but also achieving the original goal. During the hospitalization period, there was no significant difference in the incidence of postoperative complications between the two groups of patients (*P* > 0.05). After surgery, they developed fever and received anti-inflammatory agents treatment, without any wound bleeding. The control group experienced hematuria and phlegm in the throat after removing the urinary catheter, which improved after symptomatic treatment and was discharged.

**Fig. 3:**
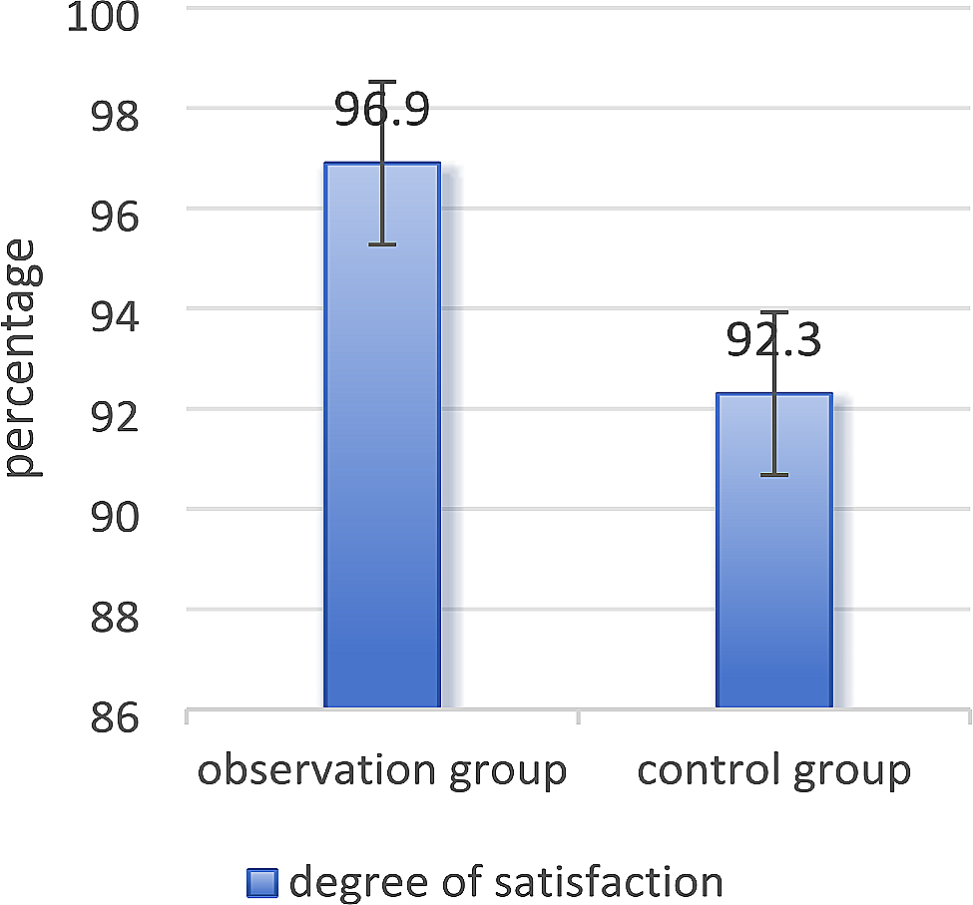
Satisfaction rate of patients in the two groups

In Table 3, the satisfaction score of the observation group was higher than the control group (*P* < 0.05). However, both groups had better medical experience, with satisfaction rates reaching over 90% (Fig. 3), and there were no complaints or dissatisfaction.In fact, the two groups of patients are consistent in implementing nursing care.However, the use of PDCA cycle management for daytime varicocelectomy has standardized the process, shortened the length of hospital stay and costs, and thus resulted in higher patient satisfaction, which is more beneficial for patients.

**Table 1:** Comparison of demographic characteristics of patients between the observation group and the control group

Variables	observation group	control group	Test statistic	p value^*^
**Numbers(n)**	65	65		
**Age**(years,m ± SD)	29.6 ± 7.7	28.7 ± 7.5	t = 0.686	0.494
**Varicocele, side**,n(%)			χ^2^ = 1.170	0.367
Unilateral	28(43.1)	22(33.8)		
Bilateral	37(56.9)	43(66.2)		
**Grade**,n(%)			χ^2^ = 3.558	0.169
I	4(6.2)	9(13.8)		
II	53(81.5)	44(67.7)		
III	8(12.3)	12(18.5)		
**BMI**(Kg/m^2^,m ± SD)	22.1 ± 3.0	22.9 ± 3.4	t=-1.552	0.123

Note:Values are presented as mean ± SD.t,t test;χ2,chi-square test

p value^*^:Comparison between the observation group and the control group

Abbreviations: BMI, body mass index. SD, standard deviation

**Table 2:** Comparison of hospitalization status of patients between the observation group and the control group

Variables	observation group	control group	Test statistic	p value^*^
**Hospital time**(day, m ± SD)	1.0 ± 0.0	3.6 ± 0.8	Z=-10.665	0.000
**Operation Time** (min, m ± SD)	72.9 ± 23.3	75.8 ± 29.3	Z=-0.159	0.874
**Cost** (yuan, m ± SD)	9826.8 ± 798.8	11586.4 ± 1482.4	Z=-7.895	0.000
Unilateral	9240.4 ± 744.7	10891.4 ± 1074.8	Z=-5.414	0.000
Bilateral	10270.5 ± 498.6	11941.9 ± 1545.5	Z=-6.769	0.000
**VAS before discharge** (score, m ± SD)	1.3 ± 0.5	1.0 ± 0.4	Z=-4.207	0.004
**Postoperative Complications**,n(%)			χ^2^ = 1.837	0.399
fever	3(4.6)	4(6.1)		
haemorrhage	0(0)	0(0)		
oscheocele	0(0)	1(1.5)		
others	0(0)	2(3.1)		

Note:Values are presented as mean ± SD.Z,nonparametric test;χ2,chi-square test

p value^*^:Comparison between the observation group and the control group

Abbreviations: VAS, visual analogue pain scale. SD, standard deviation

**Table 3:** Comparison of patient satisfaction between the observation group and the control group

Variables	observation group	control group	Test statistic	p value^*^
**grade**(score, m ± SD)	4.45 ± 0.56	4.2 ± 0.57	t = 2.496	0.014
**Proportion**,n,(%)			χ^2^ = 6.086	0.048
very satisfied	31(47.7)	18(27.7)		
satisfied	32(49.2)	42(64.6)		
average	2(3.1)	5(7.7)		

Note:Values are presented as mean ± SD.t,t test;χ2,chi-square test

p value^*^:Comparison between the observation group and the control group

## Discussion

Varicocele is a prevalent condition in urology, and it is the most common potentially treatable genital disease among men seeking infertility treatments, thereby supporting the implementation of suitable treatment strategies [17]. In clinical practice, varicocelectomy is currently the main method to solve the problem of varicocele reflux and improve fertility [1].The main surgical methods are low ligation under the microscope and high ligation under the laparoscope [18]. Laparoscopic technology has the advantages of minimal trauma, short surgical time, and can handle both sides simultaneously without changing the surgical incision. It has fast postoperative recovery and is widely used in clinical practice [19], meeting the necessary conditions for daytime surgery. While ensuring medical quality, the daytime surgery model can optimize the hospitalization process, shorten patient hospitalization time, improve bed utilization, reduce medical expenses, and reduce medical work pressure. It is a win-win model for doctors and patients [8]. Therefore, China vigorously advocates and promotes daytime surgery. At present, daytime varicocelectomy has been carried out in some centers in China and has achieved a good response. Liu et al. compared 71 daytime and inpatient patients with laparoscopic varicocelectomy [20]. The former had better hospital stays, hospitalization costs, and patient satisfaction compared to inpatients. There was no difference in surgical efficacy and postoperative complications between the two groups.The results are consistent with current study, but these studies did not standardize the management of daytime varicocelectomy. In the process of conducting many daytime surgeries, it is still necessary to continuously adjust and improve according to the characteristics of daytime surgeries themselves.

The PDCA cycle was first proposed by Dr. Deming in the 1950s [21]. It is a recognized basic method in many quality management fields. It has been adopted successively in performance management, enterprise management, schools, hospitals, and has achieved good results [22]. The PDCA cycle is applied in practical work by first identifying problems, analyzing them, developing plans, and setting goals.Then, implementation is carried out according to the plan, regularly inspections are conducted, timely feedback is provided, and the effectiveness is evaluated.Finally, goals are revised and experiences are summarized to promote continuous quality improvement [23]. These four stages can be carried out sequentially or simultaneously, and the results of the previous cycle can become a prerequisite for the new cycle [24].

Previous research has shown that the application of PDCA cycle in clinical and nursing work can help medical personnel proactively identify problems, optimize workflow [25], truly implement work, and continuously improve the quality of medical care [26]. Regular feedback and summary are conducted with patients as the core in clinical work [27], and effective experiences are summarized and saved in each cycle management to provide theoretical guidance for subsequent clinical improvement [23]. This study is based on the day surgery performed in the urology department of the Third Affiliated Hospital of Southern Medical University in the past, summarizes experience, and combines PDCA cycle to develop a standardized and personalized daytime varicocelectomy. In this mode, team members fulfill their respective responsibilities, take the patient as the center, and carry out clinical and nursing work in an orderly manner, with the general leader taking charge of the overall situation, The head nurse and deputy chief physician are responsible for managing and supervising the implementation and smoothness of each link, conducting weekly summary and feedback, continuously improving and optimizing the process.The current preliminary research data shows that the individualized management model of daytime varicocelectomy is also effective. The observation group shortened the hospitalization time, reduced hospitalization costs, improved patient satisfaction compared to the control group. However, the VAS score of the observation group before discharge was higher than that of the control group (*P* < 0.05).Possibly due to the reduction of pain in patients before discharge as the postoperative recovery time and the use of painkillers prolonged. Additionally, this study anticipates the introduction of a new laparoscopic instrument with a minimized diameter. The high-definition laparoscope measures just 5 mm, while the surgical instruments have a diameter of approximately 2.7 mm. The goal is to minimize abdominal trauma, alleviate postoperative pain, reduce VAS scores before discharge, and offer a novel strategy for the future advancement of daytime varicocelectomy by reducing instrument diameters.

Current research has several limitations. Firstly, being a single-center retrospective study, it is susceptible to potential selection bias. There may be subjective and objective factors such as unfamiliarity with the process, cognitive bias, and poor practice in diagnosis, treatment, and nursing that hinder the implementation of daytime varicocelectomy, resulting in a scarcity of analyzable data. Secondly, given the variations in patients’ geography and nationality, along with the relatively small sample size available for analysis, the analysis results may lack sufficient precision. Thus, there is a need for multi-center and multi-regional data collection to augment the sample size for further research, laying a greater foundation for clinical decision-making.

## Conclusion

In summary, daytime varicocelectomy has positive benefits for both hospitals and patients, reducing hospitalization time and cost, improving work efficiency, improving patient satisfaction, and optimizing bed utilization. The PDCA Cycle makes the daytime varicocelectomy process more reasonable and effective, further ensuring the medical quality and patient safety of daytime surgery and is worth promoting. In addition, it provides reference significance for the application of PDCA Cycle in other diseases and nursing management.

## Data Availability

All data within the manuscript are presented as part of tables or figures and have not been previously published. Additional data can be requested from the corresponding author.

## References

[1] Zavattaro M (2018). Treating varicocele in 2018: current knowledge and treatment options. J Endocrinol Invest.

[2] Verim S (2016). Prognostic predictors of fertility in young adult patients with Varicocele: Peak Retrograde Flow Velocity and Reflux Grade. J Ultrasound Med.

[3] Alsaikhan B (2016). Epidemiology of varicocele. Asian J Androl.

[4] WHO, The influence of varicocele on parameters of fertility in a large group of men presenting to infertility clinics. World Health Organization. Fertil Steril., 1992. 57(6): p. 1289-93.PMID: 1601152. 1601152

[5] Jensen C (2017). Varicocele and male infertility. Nat Rev Urol.

[6] Seiler F (2022). Laparoscopic varicocelectomy in male infertility: improvement of seminal parameters and effects on spermatogenesis. Wien Klin Wochenschr.

[7] Xue P (2022). Clinical efficacy of microscopic varicocelectomy for the treatment of Varicocele in Outpatient Day Surgery[J]. J Vascular Endovascular Surg.

[8] Bailey CR (2019). Guidelines for day-case surgery 2019: guidelines from the Association of anaesthetists and the British Association of Day surgery. Anaesthesia.

[9] Yu D, Liu X (2022). Visual analysis of the day surgery policy documents in China [J]. [in Chinese]Health Quality Management in China.

[10] Jiang L, Song Y, Ma H (2021). Future Development Vision of Day surgery in China[J]. [in Chinese] West China Journal of Medicine.

[11] Chen Q (2017). Exploration of day surgery practice in the Urology Department of Shanghai Renji Hospital[J]. Chin J Hosp Manage.

[12] Gu S (2021). Application of PDCA cycle management for postgraduate medical students during the COVID-19 pandemic. BMC Med Educ.

[13] Lu M (2022). Application of the Plan–Do–Check–act cycle in shortening the decision to delivery interval time. Risk Manag Healthc Policy.

[14] Su X, et al. To explore the application of PDCA in Hemodialysis Center and its effect on the Maintenance of Internal Fistula. Biomed Res Int. 2022;2022(p1–8). 10.1155/2022/7380632. 10.1155/2022/7380632 PMC9328984 35909478

[15] Yuan R (2017). Efficacy and safety of varicocelectomies: a meta-analysis. Syst Biol Reprod Med.

[16] Zhang GX (2017). Prospective randomized comparison of transumbilical two-port laparoscopic and conventional laparoscopic varicocele ligation. Asian J Androl.

[17] Punab M (2017). Causes of male infertility: a 9-year prospective monocentre study on 1737 patients with reduced total sperm counts. Hum Reprod.

[18] Almekaty K (2019). The role of artery-preserving varicocelectomy in subfertile men with severe oligozoospermia: a randomized controlled study. Andrology.

[19] Yu W (2016). Laparoscopic varicocelectomy in adolescents: artery ligation and artery preservation. Urology.

[20] Liu G, Wu S, He X (2018). Analysis of the effect of laparoscopic high ligation of varicocele during the day in county-level hospitals [J].[in Chinese]. J Youjiang Med Coll Nationalities.

[21] Gao Y, Chen X, Kang L (2021). The effect of Plan-Do-Check-act cycle nursing management of gynecological surgery: a systematic review and meta-analysis. Ann Palliat Med.

[22] Jin J, et al. Effect Analysis of Midwife Education and Training with PDCA model. Comput Intell Neurosci. 2022;2022(p 7397186). 10.1155/2022/7397186. 10.1155/2022/7397186 PMC9356789 35942458

[23] Pan N, Luo YY, Duan QX (2022). The influence of PDCA Cycle Management Mode on the enthusiasm, efficiency, and Teamwork ability of nurses. Biomed Res Int.

[24] Zeng F (2022). Influence of Fine Management Combined with PDCA Cycle Method on Disinfection qualified rate and performance Grade of Ophthalmic Precision instruments. Front Surg.

[25] Yao X, et al. Study on the effect of PDCA circulation method on nursing Quality Management in the Day operating room. Contrast Media Mol Imaging. 2022;2022(p 3503095). 10.1155/2022/3503095. 10.1155/2022/3503095 PMC9119782 35652037

[26] Liu C (2022). Application of the PDCA cycle for standardized nursing management in sepsis bundles. BMC Anesthesiol.

[27] Yi C et al. Study on the Influence of PDCA Cycle Nursing Based on Network Service on the Quality of Life and Nutritional Status of Hypertension Patients in Home Care. Evidence-based complementary and alternative medicine, 2021. 2021: p. 6068876-6.10.1155/2021/6068876. 10.1155/2021/6068876 PMC8553435 34721637

